# Bacterial Communities of *Ixodes scapularis* from Central Pennsylvania, USA

**DOI:** 10.3390/insects11100718

**Published:** 2020-10-20

**Authors:** Joyce Megumi Sakamoto, Gabriel Enrique Silva Diaz, Elizabeth Anne Wagner

**Affiliations:** 1 Department of Entomology, Pennsylvania State University, University Park, PA 16802, USA; eaw5308@psu.edu; 2 Calle 39 E-1 Colinas de Montecarlo, San Juan 00924, Puerto Rico; gabrielsilva@students.rossu.edu

**Keywords:** *Ixodes scapularis*, microbiome, pathogens, symbiotic bacteria, *Rickettsia*, *Rickettsiella*, *Borrelia*, *Anaplasma*

## Abstract

**Simple Summary:**

The blacklegged tick, *Ixodes scapularis*, is one of the most important arthropod vectors in the United States, most notably as the vector of the bacteria *Borrelia burgdorferi*, which causes Lyme disease. In addition to harboring pathogenic microorganisms, ticks are also populated by bacteria that do not cause disease (nonpathogens). Nonpathogenic bacteria may represent potential biological control agents. Before investigating whether nonpathogenic bacteria can be used to block pathogen transmission or manipulate tick biology, we need first to determine what bacteria are present and in what abundance. We used microbiome sequencing to compare community diversity between sexes and populations and found higher diversity in males than females. We then used PCR assays to confirm the abundance or infection frequency of select pathogenic and symbiotic bacteria. Further studies are needed to examine whether any of the identified nonpathogenic bacteria can affect tick biology or pathogen transmission.

**Abstract:**

Native microbiota represent a potential resource for biocontrol of arthropod vectors. *Ixodes scapularis* is mostly inhabited by the endosymbiotic *Rickettsia buchneri*, but the composition of bacterial communities varies with life stage, fed status, and/or geographic location. We compared bacterial community diversity among *I. scapularis* populations sampled within a small geographic range in Central Pennsylvania. We collected and extracted DNA from ticks and sequenced amplicons of the eubacterial 16S rRNA gene from individuals and pooled samples. We then used taxon-specific PCR and/or qPCR to confirm the abundance or infection frequency of select pathogenic and symbiotic bacteria. Bacterial communities were more diverse in pools of males than females and the most abundant taxon was *Rickettsia buchneri* followed by Coxiellaceae (confirmed by sequencing as an unknown *Rickettsiella* species). High *Rickettsiella* titers in pools were likely due to a few heavily infected males. We determined that the infection frequency of *Borrelia burgdorferi* ranged from 20 to 75%. Titers of *Anaplasma phagocytophilum* were significantly different between sexes. Amplicon-based bacterial 16S sequencing is a powerful tool for establishing the baseline community diversity and focusing hypotheses for targeted experiments, but care should be taken not to overinterpret data based on too few individuals. We identified intracellular bacterial candidates that may be useful as targets for manipulation.

## 1. Introduction

Ticks are obligately hematophagous arachnids that are found worldwide parasitizing vertebrates. In the United States, the blacklegged tick, *Ixodes scapularis*, transmits the pathogens or parasites that cause Lyme disease (*Borrelia burgdorferi),* babesiosis, anaplasmosis, ehrlichiosis, and Powassan encephalitis [1]. In addition to pathogens and parasites, *I. scapularis* is also populated by several other nonpathogenic microorganisms, some of which might be targets for tick control or blocking pathogen transmission. 

In order to investigate questions about tick bacteria and interbacterial interactions within (e.g., potential impacts of native microbiota on pathogen transmission), we need to know what is present. Microbiome sequencing (sequencing of eubacterial 16S rRNA amplicons) represents a powerful way to assess microbial variation at the individual and population levels (through sample pooling). In ticks, next generation sequencing platforms offer deeper coverage of the bacterial community, identification of unculturable organisms, and detection of rare taxa [2,3].

One potential application of tick microbiome research is in identifying candidates for applied manipulation. Bacteria (including obligate endosymbionts) are attractive targets for non-chemical vector control strategies. Microbiota (bacteria in particular) may modulate the invasion, replication, and/or transmission of pathogens in vector arthropods or potentially inhibit transmission to vertebrate hosts [4,5,6]. In some systems obligate intracellular endosymbiotic bacteria can influence the biology of the arthropods themselves, altering life history characteristics positively or negatively [7,8,9].

In numerous *I. scapularis* microbiome studies over the last two decades, the dominant bacterial taxon identified was *Rickettsia* [3,10,11,12]. Bacterial community composition apart from this symbiont, however, varied depending on life stage, sex, fed status, and/or geographic location [3,12,13]. Adult bacterial diversity is less compared to immature stages, indicating that the remaining bacterial taxa represent the stable native community members [13,14]. Thus, bacterial community differences may represent locality-specific microbiomes. 

In our study we wanted to know the bacterial variability between sexes and populations of blacklegged ticks from Central Pennsylvania. We sequenced amplicons of the eubacterial 16S rRNA to (1) determine the baseline bacterial diversity from ticks collected from within a relatively small geographic area, (2) confirm the species identity of key taxa using taxon-specific PCR and Sanger sequencing, and (3) estimate the relative abundance of key bacterial taxa by PCR and/or qPCR in pooled DNA and individual ticks collected from central Pennsylvania. 

## 2. Materials and Methods

### 2.1. Sample Collections

**Figure 1:**
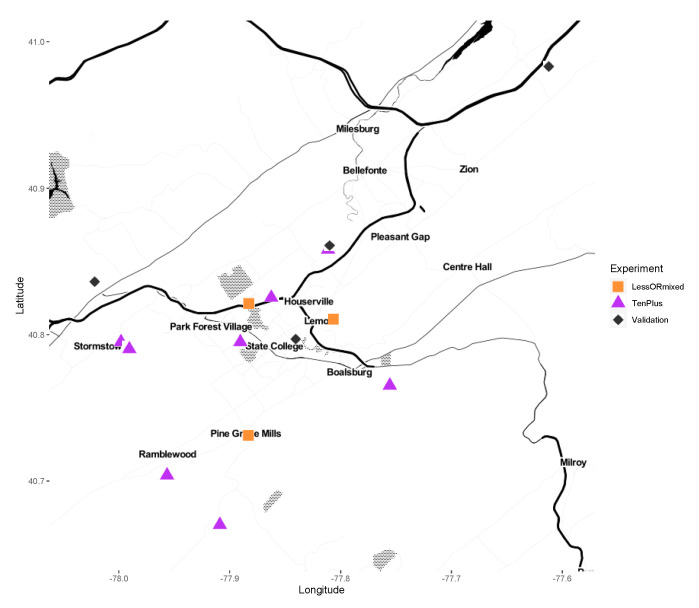
Field collection sites. Squares (“LessORMixed”) represent pools containing less than 10 individual ticks, mixed pools of male and female, or both. Triangles (“TenPlus”) represent pools containing 10 or more individuals. Diamonds (“Validation”) represent collection sites assayed by qPCR.

Adult male and female *I. scapularis* were collected from Central Pennsylvania from 2012 to 2019. The collection sites were between 0.804 and 43.13 km (0.5–26 miles) apart (Figure 1). Host-seeking adults were collected with a drag cloth (36′ × 45′; 91.44 cm × 114.3 cm). Samples were stored alive in 20 mL scintillation vials until returned to the laboratory for immediate surface sterilization by washing in 70% ethanol for 15 s, then 1 min in 10% bleach, followed by three sequential washes in autoclaved, nuclease-free water, dried on autoclave-sterilized filter paper, and stored at −80 °C until processed for DNA extraction. Samples were sorted to sex and species confirmed before extraction [15]. 

### 2.2. DNA Extraction

For preliminary 16S rRNA sequencing, genomic DNA was extracted from 30 individual adult ticks collected in 2012 (Table 1). DNA from Shaver’s Creek (2012) was not sequenced because the DNA quality was too low. Each sample was surface-sterilized as described above and bisected longitudinally with a new flame-sterilized razor blade. DNA was extracted from individual ticks using the DNeasy Blood and Tissue kit (Qiagen, Germantown, MD). Samples were submitted for 16S rRNA sequencing of the hypervariable region V6 on the Illumina MiSeq platform to assess the baseline species composition of field-collected blacklegged ticks. After the preliminary sequencing run on individual tick DNA, we added more populations, but used pooled DNA from individually extracted ticks due to budget constraints. This approach allowed us to sequence results from the pools to then target specific taxa and determine infection frequency or titer. 

We extracted DNA from 298 individual ticks (collected in the period 2013–2014). DNA was pooled from the individuals by sex and population (16 pools) and submitted for paired-end Illumina MiSeq sequencing (Table 1). Each sample was surface-sterilized and processed as above. One half was used for extraction using the DNeasy Blood and Tissue kit (Qiagen), while the other half was archived at −80 °C. 

An additional 3 pools (from 31 ticks) were also submitted for sequencing. The DNA samples were extracted pooled ticks (i.e., not individually extracted) and represented either mixed samples of males and females or populations with less than 10 individuals. These ticks were surface-sterilized and extracted as described above, but were not bisected individually, nor were they archived.

For comparison of geographically distant populations, we also submitted for sequencing two pools of extracted DNA from individual female ticks representing two distinct mitochondrial clades from Mississippi described previously [16], (Table 1). These ticks had been previously extracted individually using the GenElute Bacterial DNA extraction kit (#NA2110, Millipore Sigma, St. Louis, MO, USA).

### 2.3. Sample Preparation for Sequencing

Genomic DNA for each tick was quantified with a Nanodrop spectrophotometer and adjusted to at least 5 ng/µL. Equal volumes of adjusted DNA were pooled by population and sex and submitted for sequencing (for a total of 16 submitted pools). The extracted genomic DNA was submitted for Illumina MiSeq sequencing using primers to amplify either the V6 or V4 hypervariable region of the bacterial 16S rRNA gene region.

### 2.4. Analysis of Microbiome Sequence Data

Sequences were aligned, filtered to remove chimeric sequences, and analyzed using Dada2, Phyloseq, and RStudio (1.3.1073). Data were visualized using the R packages Phyloseq, Microbiome, and ggpubr [17,18,19,20]. Reads were assigned taxonomic identity using the Dada2 taxonomy assigner and either the Greengenes or the Silva (v128) reference database of eubacterial 16S rRNA [21,22]. Shannon and inverse Simpson indices were used for measuring richness and evenness. Statistical comparisons between groups were performed using the Kruskal Wallis test. Community dissimilarity (Bray–Curtis index) was evaluated between groups. Principal coordinate analyses (PCoA) were calculated and plotted to visualize bacterial community structure between groups using Phyloseq. Statistical comparison between groups was performed to run permutational multivariate analyses of variance (PERMANOVA using 999 permutations). The significance level was set to 0.05. 

### 2.5. Taxon-Specific Amplification and Sequence Confirmation

We used taxon-specific primers (either previously published or designed for this study) on a subset of tick samples positive for *Rickettsia*, *Rickettsiella*, *Borrelia,* and *Cardinium* (Table 2 and Table 3). We also assayed the pooled DNA with PCR specific to *Wolbachia*, nematode or chalcid wasp genes (Table 2 and Table 3) but did not sequence these amplicons. Amplicons were separated on a 0.5X TAE-2% agarose gel. Bands were excised and purified for cloning with the StrataClone PCR Cloning kit (#240205, Agilent, Santa Clara, CA, USA), following manufacturer’s guidelines. Plasmids were purified using the E.Z.N.A. Plasmid mini kit (Omega Biotek #D6942) and sequenced in both directions on an Applied Biosystems (ABI) 3130/Genetic Analyzer. Sequences were compared to known sequences in the Genbank NR nucleotide database.

Sequences were aligned, trimmed, and phylogenetically analyzed in MEGA7 [34]. The evolutionary history was inferred by using the Maximum Likelihood method based on the General Time Reversible model [35]. Bootstrap consensus was inferred from 1000 replicates and branches in fewer than 50% bootstrap replicates were collapsed [36]. Genbank accession numbers used for each taxon/gene analysis are listed in Table 4. 

### 2.6. Assays for Tick-Borne Pathogens

#### 2.6.1. *Borrelia burgdorferi* Infection Frequency

Sex-specific infection frequencies from each population were assessed by amplifying the *Borrelia burgdorferi* OspC gene (non-nested PCR) and the flagellin FlaB gene (nested PCR) following conditions described previously [24,25] (Table 2 and Table 3). DNA extracted from individual ticks was tested from each of the pooled populations and the percentage of infected individuals per total pool (sex–location) was calculated. 

#### 2.6.2. Taqman PCR Assay for *Anaplasma phagocytophilum*

We used a Taqman assay for detection of *Anaplasma phagocytophilum* [23], with the following modifications: we used a PrimeTime^®^ Standard qPCR Assay (in which primers and probes are mixed and received in a single tube) (Integrated DNA Technologies, Inc., Coralville, IA, USA) and the probe had a double-quenching Zen/Iowa Black instead of tetramethyl rhodamine (TAMRA) quencher. Relative titer for each of gene was compared to the reference gene *I. scapularis* actin gene. Reactions were run for 40 cycles × (95 °C/15 s, 60 °C/60 s). Each sample was run in triplicate.

### 2.7. Taxon-Specific Quantitative PCR of Nonpathogenic Symbiotic Bacteria

Taxon-specific qPCR primers were used to assess relative titer (Table 3) on individuals or on pools containing at least 10 individuals or more per sex–population pool. In addition to running assays on pools from which we had Illumina sequence data, we tested the *Rickettsia* and *A. phagocytophilum* qPCR assays on pooled DNA from 232 ticks (10 pools of males or females) collected from other locations and/or from other years (Table 3). 

qPCR primers were designed for *Rickettsia*, *Flavobacterium*, *Pseudomonas*, and *Rickettsiella* using the National Center for Biotechnology Information (NCBI) tool Primer-blast [37] and confirmed to match the desired taxa by Sanger sequencing of the amplicons. PCR mixes were made with the PerfeCTa SYBR Green FastMix (Quanta Bioscience, Inc., Gaithersburg, MD, USA). All reactions were run on a Rotor-Gene Q 5plex HRM System (QIAGEN) for 40 cycles × (95 °C/10 s, 60 °C/15 s, 72 °C/15 s). Relative titer for each gene was compared to the reference gene *I. scapularis* actin gene (Genbank accession # XM_029983741.1). Assays run before v4 sequencing (on *Rickettsia*, *Flavobacterium*, and *Pseudomonas* against Actin) were run in duplicate. After v4 sequencing, all qPCRs were run in triplicate (*Rickettsia*, *Rickettsiella*, and actin). 

## 3. Results

### 3.1. Sequencing of Individual and Pooled Tick DNA Using V6 Hypervariable Region

We originally chose to sequence the V6 region because at the time we thought it was better suited for genus and species-level resolution. We had Illumina data on 30 individuals from two populations but found that the amount of variability from one pool of females (Shaver’s Creek) was very different from the females from another pool (Big Hollow) and more closely matched that of the males (Supplementary Figure S1). We could not evaluate the effect of sex or population on microbial diversity with the limited number of samples and with only one pool of males. We observed that the most abundant taxa were Rickettsiaceae, and Pseudomonadaceae (Supplementary Figure S1). When evaluating the titers of *Rickettsia*, *Pseudomonas*, and *Flavobacterium* by qPCR, we found the titers of the latter two taxa to be considerably less than indicated by sequence counts. We did detect a significant sex-specific difference in these three taxa by qPCR (Supplementary Figure S2). 

As the microbiome sequencing of individual ticks represented only two populations, we revisited these populations to collect fresh ticks for the pooled sequencing run in addition to six new populations. We found that Pseudomonadaceae dominated not only every one of our pools but also that of every sample in the same sequencing run (from unrelated experiments). We suspected that there was a V6 primer bias and we re-sequenced the V4 region of the same pools for subsequent analyses (below). We also found that Greengenes was not an appropriate reference database for environmental data and ran subsequent taxonomic assignments with the Silva database. 

### 3.2. Bacterial Community Composition of Pooled Tick DNA Using the V4 Hypervariable Region

We compared populations of variable size and location and found that: (1) the bacterial taxon found in every population was *Rickettsia* (Family Rickettsiaceae) and (2) there was a significant difference in bacterial community diversity between geographically distant populations (Mississippi versus Pennsylvania) (PERMANOVA: 999 permutations, *p* = 0.047). After assessing the sequencing results from the individual data, we concluded that while low sample size populations (less than 10 samples per pool) were useful for confirming that *Rickettsia* was present, they might not accurately reflect the bacterial diversity within populations. We also could not evaluate the effect of sex in pools containing both sexes. We therefore focused all subsequent analyses on pools of ticks from Pennsylvania with at least 10 samples and which contained only male or only female ticks. We further determined that one female pool from the Ag Progress Days population (“APD”) was an outlier (Grubb’s outlier test”, Z-value 2.734, *p* < 0.05) and removed it from subsequent analyses (Supplementary Figure S3).

**Figure 2:**
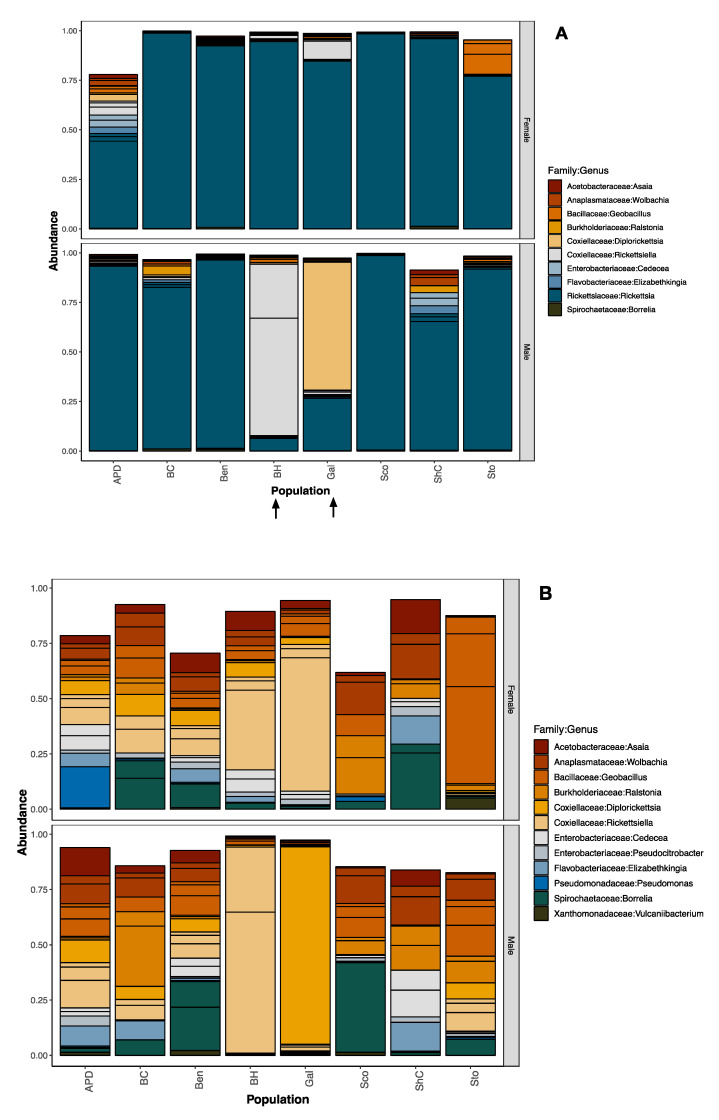
Relative read abundance of 16S rRNA (hypervariable region v4) sequenced from pools from PA. Plots show the most abundant bacterial families sorted by population and sex. Pools shown have more than 10 individuals and only male or only female ticks. (**A**) With Rickettsiaceae. (**B**) Without Rickettsiaceae. The taxonomic assignment was performed using the Silva (v128) reference database. Populations: APD = Ag Progress Days site, BC = Blue Course, Ben = Benner Springs, BH = Big Hollow, Gal = Galbraith, Sco = Scotia/State Gameland 176, ShC = Shaver’s Creek, Sto = Stormstown.

The most abundant bacterial families identified in the ticks we tested were Acetobacteriaceae, Anaplasmataceae, Aeromonadaceae, Bacillaceae, Burkholderiaceae, Coxiellaceae, Enterobacteriaceae, Flavobacteriaceae, Rickettsiaceae, and Spirochaetaceae (Figure 2A, Supplementary Table S1). As expected, the most abundant bacterial taxon was in the family Rickettsiaceae (although there were four distinct amplicon sequence variants), but the next most abundant taxa matched two genera within the family Coxiellaceae (*Rickettsiella/Diplorickettsia*). To see the other less-abundant taxa, we removed Rickettsiaceae for visualization purposes (Figure 2B), but not from downstream analyses. 

### 3.3. Sex- and Location-Specific Differences in Microbial Diversity

**Figure 3:**
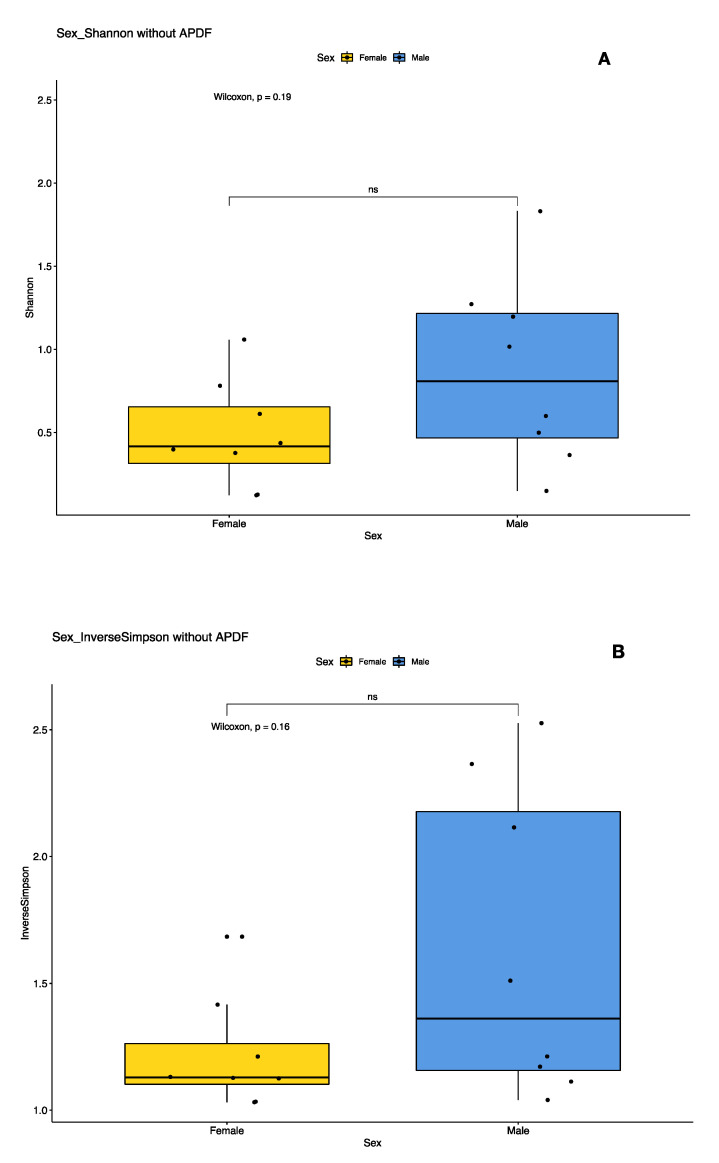
Plots of Alpha diversity indices of female versus male PA populations with at least 10 individuals. Plot (**A**) represents the Shannon diversity index and plot (**B**) represents the Inverse Simpson diversity index. The spread of indices between male pools is greater than the spread between female pools, but not significantly.

**Figure 4:**
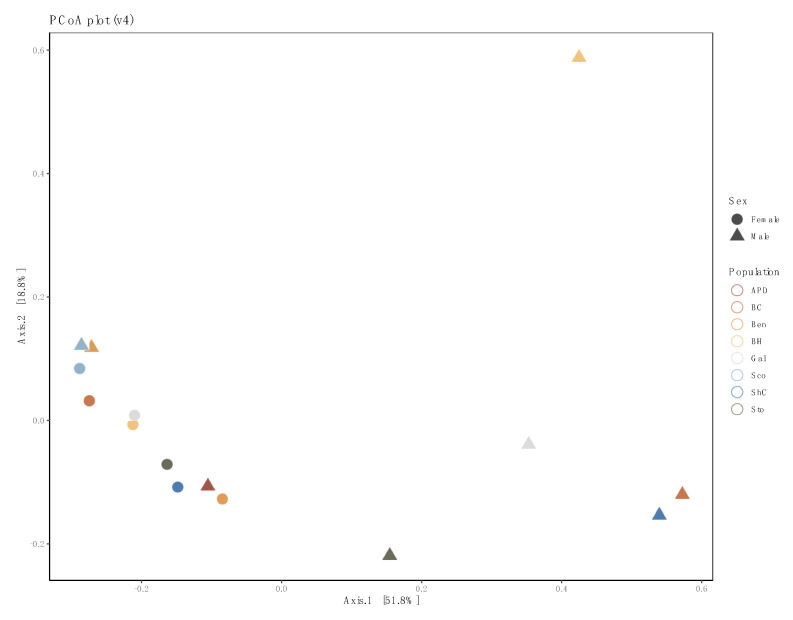
Principal coordinates analysis (PCoA) plot of 16S rRNA data from pooled samples of sample-based ecological distance. Colors represent populations, while shapes indicate sex. Female pools cluster together while male pools were more diverse.

We examined the effects of sex and population on the bacterial diversity of pooled samples. Diversity indices suggest that female pools clustered tightly, while male pool diversity was more diffused (variable), but not significantly so (Figure 3). In the principal coordinates analysis, much of the variability could be accounted for across two axes (51.8% × 18.8%) (Figure 4). We did not detect a significant difference in bacterial community between locations (PERMANOVA, 999 permutations *p* = 0.771), but did detect a significant difference between sexes (PERMANOVA, 999 permutations, *p* = 0.022).

### 3.4. Confirmation by Gene-Specific Amplification and Sequencing

In all individuals and pools, we found the most abundant taxon (with two exceptions) was an alpha-proteobacterium in the family Rickettsiaceae, genus *Rickettsia.* Fragments from each of these pools were amplified with taxon-specific primers, sequenced, and aligned. We determined that the *Rickettsia* outer membrane rompA fragments blast aligned to the symbiotic species *Rickettsia buchneri* at 99.56–99.81% identity across 100% of the 1595 bp fragment. Phylogenetic comparison further supported that these fragments were clustered with *R. buchneri* (Supplementary Figure S4).

In two populations (MB007 and MB010, corresponding to pools of males from Big Hollow and Galbraith, respectively), the dominant reads matched the genera *Diplorickettsia* and *Rickettsiella* (gamma-proteobacterial family Coxiellaceae). To confirm the identity, we sequenced fragments of the rpoB gene using primers to the Order *Legionellales* and found the closest match for MB007 was to *Rickettsiella melolonthae* (89.85% of a 570 bp fragment) and the closest match for MB010 was *R. viridis* (81.50% of 575 bp). Using a maximum likelihood analysis of similar sequences, we found that both sequences clustered with *R. viridis* and *Rickettsiella* sequences from other arthropods including ticks (Supplementary Figure S5). 

A 1075 bp fragment of the *Cardinium* 16S rRNA gene was amplified, sequenced, and confirmed to match a *Cardinium* found in an *I. scapularis* cell line (accession # AB001518.1 at 99.81%. Using a maximum likelihood analysis of similar sequences, we found the sequences clustered with *Cardinium* from other arthropods including the aforementioned cell line (Supplementary Figure S6).

### 3.5. Confirmation of Borrelia burgdorferi Identification and Frequency in Ticks Tested

**Figure 5:**
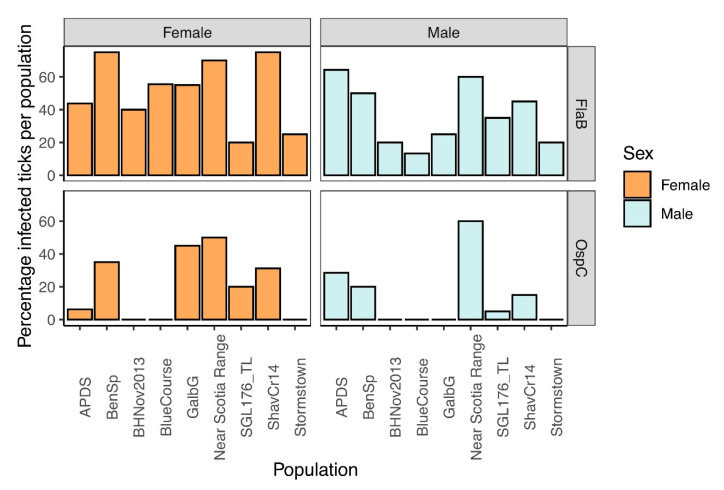
*Borrelia burgdorferi* infection frequency of individual ticks. Top two plots represents *B. burgdorferi* infection frequency of individuals within each pool of male or female ticks by FlaB. The bottom two charts represents *B. burgdorferi* infection frequency (%) of individuals within each pool of male or female ticks by OspC.3.5. Relative abundance of key taxa by sex and population.

The 16S rRNA sequencing results of the pools indicated that all pools were infected with an Amplicon sequence variant (ASV) matching the genus *Borrelia*. We confirmed the identity of *B. burgdorferi* sensu stricto by amplifying and sequencing fragments with *B. burgdorferi*-specific primers (OspC and FlaB). Sequences for OspC (333 bp) matched *B. burgdorferi* WI91-23 plasmid WI91-23_cp26 (accession # CP001446) at 100% identity. The FlaB sequences (466 bp) matched *Borrelia burgdorferi* strain B31_NRZ (accession # CP019767.1) at 100% identity. The frequency of infected individuals per population was between 20 and 75% (Figure 5). There was no significant difference in *B. burgdorferi* abundance between male and female adult ticks: OspC (Mann–Whitney U = 31.5, z-score = 0.751, *p*-value = 0.453) or FlaB (Mann–Whitney U = 24.5, z-score = 1.369, *p*-value = 0.171).

### 3.6. Taxon-Specific Quantitative PCR

**Figure 6:**
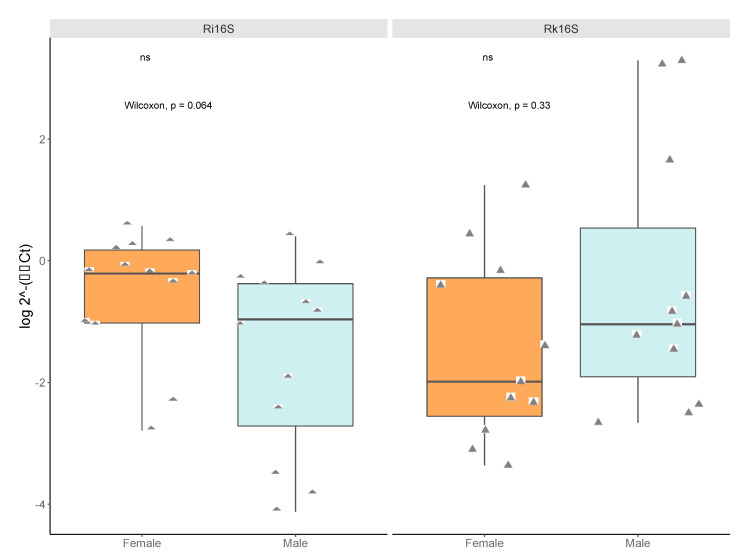
Relative titers of bacterial 16S rRNA gene from *Rickettsia* and *Rickettsiella* between pools of male and female ticks (2^−(∆∆Ct)^). Data were normalized to a housekeeping gene *Ixodes scapularis* actin. Diamonds represent male or female pools tested (pools submitted for sequencing plus pools from “JulianF2015”, “BenSpF2017”, “BenSpF2018”, “HowardF” and “LedererF2019”). For *Rickettsiella*, the pools for “JulianF2015“ or “HowardF“ did not amplify and were not included in the analysis. There was no significant sex difference in *Rickettsia* (qRi16S) titers or *Rickettsiella* (qRk16S) titers. Statistical significance was determined by Mann–Whitney U and labels were produced in R.

We compared titers of *Rickettsia* and *Rickettsiella* by qPCR of pooled DNA sent for sequencing, additional pools used for validation, and the individuals from two pools of males with high *Rickettsiella* read counts (15 males from Big Hollow/MB007 and 20 males from Galbraith/MB010), and from 20 individual females from a population with low titers of *Rickettsiella* (Shaver’s Creek/MB013). No significant titer differences for *Rickettsia* nor *Rickettsiella* were detected between sexes in pooled DNA (*Rickettsia* two-tailed Mann–Whitney U = 43.5, z-score = 1.85, *p*-value = 0.064; *Rickettsiella* Mann–Whitney U = 45, z-score = −0.985, *p*-value = 0.327) (Figure 6). 

When we assayed individuals from select pools of males or females, we found that *Rickettsia* titers were significantly higher in individual females versus males (Supplementary Figure S7) (Mann–Whitney U = 90.5, z-score = −4.53156, *p*-value is <0.00001), while *Rickettsiella* titers in individual females were significantly lower than males (Mann–Whitney U = 14.5, z-score = 5.86128, *p*-value is <0.00001). Within male pools, a few individual males had extremely high titers (several folds higher than others) that may account for the high read abundance of *Rickettsiella* observed in pools submitted for Illumina sequencing (Supplementary Figure S7). 

We identified ASVs matching the genera Candidatus *Cardinium* and *Anaplasma phagocytophilum* at low frequencies. *Cardinium* is a known symbiotic bacterium of other arthropods in the Bacteroidetes so, although the read counts for Candidatus *Cardinium* were below the threshold (not within the top 20 most abundant taxa), we chose to confirm its identity using genus-specific amplification and Sanger sequencing. In all pools assayed, only a single population matched *Cardinium.* Further screening of each individual within that population identified that only a single individual in the female pool of 20 tested positive. 

**Figure 7:**
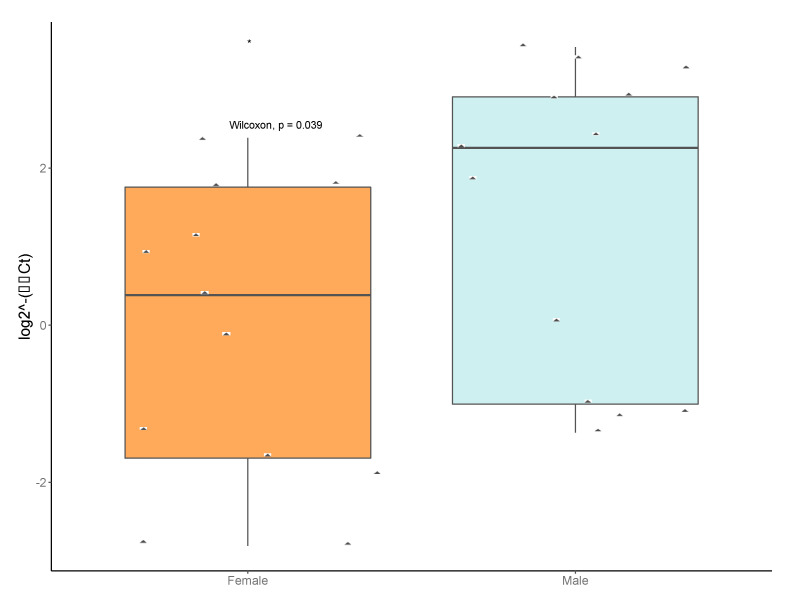
Relative titers of *Anaplasma phagocytophilum* MSP2 gene from pools of male and female ticks. Data were normalized to a housekeeping gene *Ixodes scapularis* actin. Diamonds represent male or female pools tested (pools submitted for sequencing plus pools from “JulianF2015”, “BenSpF2017”, “BenSpF2018”, “HowardF” and “LedererF2019”). Male pools had significantly higher titers of Anaplasma phagocytophilum (*). Statistical significance was determined by Mann–Whitney U and labels were produced in R.

We confirmed the presence and relative titers of *Anaplasma phagocytophilum* in our pools. Additionally, we tested 10 pools sorted by sex from four additional locations: Julian, PA (2015); SGL333 (2017 and 2018), Howard, PA (2019), and Lederer Park (2019). The ApMSP2 levels were significantly higher in male versus female tick pools (Figure 7).

Sequencing results suggested that *Wolbachia* was present in almost all pools. We did not investigate the infection frequency or titers in pools, but we recognized that *Wolbachia* could be present as a symbiont of an endoparasite of the tick. We therefore tested for presence of wasp or nematode DNA but did not detect either. 

## 4. Discussion

Tick obligate intracellular symbionts have been closely studied as potential tools for manipulation of ticks, tick-borne pathogens, or both. The first record of symbiont–pathogen antagonism in ticks was observed in populations of the wood tick *Dermacentor andersoni* from east and west sides of the Bitterroot Valley in Montana [38]. It was hypothesized that the nonpathogenic east-side agent (*R. peacockii)* could competitively exclude the highly pathogenic *R. rickettsii* from tick ovarial tissue [38]. Subsequent isolation and characterization of *R. peacockii* revealed that it was genetically similar to the highly virulent *R. rickettsii*, but contained several insertion sequences throughout its genome [39,40]. Interference was also observed between *R. montanensis* (formerly known as *R. montana*) and *R. rhipicephali,* and between different strains of *Anaplasma marginale* [41,42]. As yet, no evidence of competitive exclusion has been described in *Ixodes scapularis.*

Our study used tick microbiome sequencing not as an endpoint, but more as a scouting expedition into what taxa were common and which were uncommon or rare. We then followed up with assays on infection frequency or abundance. As microbiome data are not always reliable below the family or genus level, we used taxon-specific primers, sequenced the amplicons and phylogenetically confirmed the identity of these taxa to determine what species (or isolates) were present. 

Bacterial diversity diminishes over the lifespan of a tick and so bacterial taxa identified in adult ticks were either acquired post-molting or retained from the previous life stage [14]. However, our study did not include immature stages so we cannot address ontological differences in bacterial community composition from our populations. We do know that at least some of these taxa were probably stable infections, as these taxa are known to be vertically transmitted. As expected, *R. buchneri* was present in all samples tested. *R. buchneri* is, one of many nonpathogenic spotted fever group rickettsias, contains several genes that may supplement an incomplete heme biosynthesis pathway in *I. scapularis* [43]. However, it is not known whether *R. buchneri* is essential for tick survival, plays a substantial role in defining in the microbial community, or interacts in some way with invading pathogens.

Another vertically transmitted taxon was the second most abundant family Coxiellaceae. Most pools contained reads matching the genus *Rickettsiella* but reads from one male pool (GalM) were bioinformatically identified as *Diplorickettsia*. For this study, we chose to use the NCBI taxonomy, which lists both genera under Legionellales: Coxiellaceae. However, we did not detect *Diplorickettsia* when we sequenced amplicons using Legionellales-specific primers. *Rickettsiella* has been described in *I. scapularis* from Massachusetts, and in other *Ixodes* species [12,44]. To date, *I. scapularis*-associated *Rickettsiella* is not known to be pathogenic to humans, although in invertebrate hosts there are both pathogenic and mutualistic isolates [45]. It is not known if the *I. scapularis Rickettsiella* interacts with other intracellular organisms (e.g., *R. buchneri*). However, *Rickettsiella* isolate in *I. woodi* resides within Malpighian tubules and ovaries, so there is a possibility of interbacterial interaction [44]. *Rickettsiella* in *I. woodi* was hypothesized to be involved in the parthenogenic nature of one lab colony [44,46]. Presumably the *Rickettsiella* in *I. scapularis* does not cause parthenogenesis since we detected this genus in both males and females from field populations. We are investigating what, if any, effect *Rickettsiella* may have on *I. scapularis* biology.

We also detected *Wolbachia*, an alpha-proteobacterial intracellular symbiont that infects a wide range of invertebrate hosts. *Wolbachia* can have little effect on its host or can alter its reproductive biology in different ways ranging from male-killing, parthenogenesis, or cytoplasmic incompatibility [47,48,49]. While this was not our experimental focus, it was an interesting observation given that *Wolbachia* has been found to positively or negatively affect pathogen transmission in other vector systems [5]. *Wolbachia* has been found in other *I. scapularis* studies [10,12], but it has not been determined whether the ticks themselves are infected or if they are parasitized by something that is itself infected with *Wolbachia*. 

We also tested for presence of wasps and nematodes and found no evidence of either in our populations. One important caveat is that we only tested adult ticks. *Ixodiphagus* wasps are known to infect immature life stages and are presumed to be fatal (hence, adults should not have any wasp DNA). However, it is conceivable that some parasitized ticks could clear the infestation and retain residual *Wolbachia* and/or wasp DNA. A more in-depth study of all life stages would be necessary to conclusively determine (1) if ticks in the tested populations were parasitized by Ixodiphagus wasps and (2) that the *Wolbachia* identified is due to an infection of an endoparasite infecting the ticks. It would be interesting to determine if endoparasites are present and if so, how prevalent they are in *I. scapularis* populations. 

We examined two pathogenic bacterial taxa found in our sequencing results. All sex–population pools contained reads corresponding to the Spirochaetaceae matching the genus *Borrelia. Borrelia miyamotoi* has been shown to occur at low frequencies in some populations of *I. scapularis* [50], but we did not investigate this and only assessed the presence of *B. burgdorferi*. We found that 20–75% of individual ticks were infected with *B. burgdorferi* but did not detect a sex-specific pattern. In contrast, the other pathogenic taxon we examined, *Anaplasma phagocytophilum*, was statistically higher in titer in males than female pools. It is unclear what the biological significance, if any, of this would be. Males are not considered epidemiologically important as vectors. However, if they were infected, they represent infected individuals that acquired infections either as larvae or nymphs. Cases of reported human anaplasmosis cases increased during the summer months, coincident with the active period of *I. scapularis* larvae and nymphs (https://www.cdc.gov/anaplasmosis/stats/index.html). Infected adult ticks, therefore, reflect the presence of the pathogen circulating in the tick populations in the previous year. 

We explored the use of pooled sequencing as an initial assessment of the microbial community composition at the population level. The advantage of using this approach is twofold. First, we can return to archived samples for additional studies. Second, we minimize the number of samples initially submitted for sequencing to identify populations of interest for further individual-level screening. One obvious problem with this approach, though, was that individuals with high infection loads of taxa of interest might skew the results, so this needed to be confirmed through assays of individuals. 

The pooled approach allowed us to find a rare *Cardinium* symbiont that we confirmed to be from only one of 40 individuals tested from a single population. To our knowledge, this taxon has not been found in other next-generation sequencing-based microbiome studies, presumably because it is so rare. We do not know how widely this *Cardinium* species is distributed, nor what its effect, if any, on its host. The first published account of the *I. scapularis*-associated *Cardinium* (then referred to as the “*Cytophaga*-like organism”) was from ticks collected in Nantucket MA where it was extracted from ticks and grown in tick cells [51]. It remains to be seen whether it is a transient introduction into the local tick population (e.g., from a tick transported on a migrating bird or mammal), whether it is affected in some way by the native microbiota, or whether it has any impact on the tick host. *Cardinium* is an intracellular bacterium known to be associated with ovaries and midguts of arthropods, so it is conceivable that it may interact with other intracellular bacteria, pathogenic or otherwise [52].

We observed several limitations of relying on Illumina data alone without a biological context. First, we realize that the number of individuals tested was relatively small for some of the populations. This was a reflection of the abundance in sampled areas, but a few individuals with heavy infections of one or more taxa can skew the overall outcomes, resulting in a misleading conclusion. Second, we found that populations that were close together geographically could have very different microbial communities (either containing unique taxa or very different abundances of taxa). Had we randomly selected one or two populations for comparison with other populations in another geographic location (e.g., another state), we might conclude that these two populations represented the diversity from the state of Pennsylvania. This would have been inaccurate and missed key taxa. Lastly, choosing the right hypervariable region and correct reference database can profoundly affect the results. In our case, this resulted in a significant setback that included re-sequencing, reanalysis, and qPCR validation. Had we not also validated these data by qPCR, we would have made an erroneous interpretation of the dominant taxa and the potential implications of tick bacterial community dynamics. Thus, although next-generation sequencing allows researchers to obtain a deeper depth of coverage, it does not account for unknown biases (V6 was not known to be biased in 2013, when the sequencing was initiated).

It is important to remember that, while bacterial 16S sequencing is a powerful tool for exploring taxa present in a given study, it is merely a basis for generating hypotheses and should not be relied upon to extrapolate conclusions without subsequent validation of infection frequencies. We targeted specific taxa from pools or individual tick DNA using the individual tick data to determine titers or infection frequencies. With the caveat that our data represent a small sample size, our data suggest that the microbial population dynamics can be highly variable among individuals in the same population, and between populations that are, at most, 26 miles apart. Thus, given that diversity at the local scale is so variable, patterns between and across large geographic areas should be considered suspect without sufficient sampling of each chosen population and across the collection range. 

## 5. Conclusions

We have a better understanding of what the bacterial community composition is of the local tick population. While we investigated only a few of the taxa that we observed in detail, there are many more bacteria whose presence raises interesting questions. We still lack an understanding of how (or if) a succeeding dominant taxon can cause the reduction in a native rickettsial symbiont (e.g., through direct competition for resources or receptors or indirectly inducing host immunity or production of toxins that inhibit rickettsial intracellular growth). Why are only some individuals in certain populations or sex more heavily infected with *Rickettsiella* than *R. buchneri*? Is there direct interaction between microbes that influences the within-tick microbial community composition? Are the microbial communities pre-determined by the mother or is there any transmission of bacteria from the males to offspring? Are there bacteria transmitted from the mother to the offspring by other means (e.g., egg smearing)? From the perspective of tick control, the question remains: Could any of these taxa be targeted for control or alternation of pathogen transmission? We hope to answer some of these questions in future studies.

## Figures and Tables

**Table 1:** Collection information for tick samples sequenced on the Illumina MiSeq platform. All samples were collected from the wild and DNA was extracted from individual ticks unless otherwise specified. DNA was then submitted for sequencing from “Individuals” or “Pooled” from individually extracted ticks. DNA previously extracted from individual females from Mississippi were pooled and included for comparison with distant populations. Samples were submitted for sequencing individually or as pools of DNA extracted from individuals. Sequenced hypervariable regions of the bacterial 16S rRNA gene are noted.

Collection Year	Sample ID	Population	Sex	Individuals or Pools Sequenced (N)	Region Sequenced (V4 or V6)
2012	BHF01-F10	Big Hollow (BH)	Females	Individuals (10)	V6
2012	BHM01-M10	Big Hollow (BH)	Males	Individuals (10)	V6
2012	SCF01-F10	Shaver’s Creek (ShCr) ª	Females	Individuals (10)	V6
2013	MB001	Stormstown (Sto)	Females	Pooled (20)	V4/V6
2013	MB002	Stormstown (Sto)	Males	Pooled (20)	V4/V6
2013	MB004	Blue Course (BC)	Females	Pooled (18)	V4/V6
2013	MB003	Blue Course (BC)	Males	Pooled (15)	V4/V6
2013	MB006	AgProgressDays (APD)	Females	Pooled (16)	V4/V6
2013	MB005	AgProgressDays (APD)	Males	Pooled (14)	V4/V6
2013	MB008	Big Hollow (BH)	Females	Pooled (20)	V4/V6
2013	MB007	Big Hollow (BH)	Males	Pooled (15)	V4/V6
2013	MB009	Galbraith (Gal)	Females	Pooled (20)	V4/V6
2013	MB010	Galbraith (Gal)	Males	Pooled (20)	V4/V6
2013	MB012	Benner Springs (Ben)	Females	Pooled (20)	V4/V6
2013	MB011	Benner Springs (Ben	Males	Pooled (20)	V4/V6
2013	MB013	State Gameland 176 (SGL176/Sco)	Females	Pooled (20)	V4/V6
2013	MB014	State Gameland 176 (SGL176/Sco)	Males	Pooled (20)	V4/V6
2014	MB015	Shaver’s Creek (ShCr)	Females	Pooled (20)	V4/V6
2014	MB016	Shaver’s Creek (ShCr)	Males	Pooled (20)	V4/V6
2014	Lem	Lemont ^b^	Females	Pooled (3F)	V4/V6
2014	PGM	Pine Grove Mills ^b^	Mixed	Pooled (4F, 4M)	V4/V6
2014	Toft	Toftrees ^b^	Mixed	Pooled (11F, 9M)	V4/V6
2009	MS1	Mississippi clade 1 (F3, F5, G4, G6) ^c^	Females	Pooled (4)	V4/V6
2009	MS2	Mississippi clade 2 (F6, F8, G11) ^d^	Females	Pooled (3)	V4/V6

ª Individual males from this population/sampling time did not have high enough DNA quality for sequencing. ^b^ DNA from these locations was extracted from pooled samples, not extracted from individual samples. ^c^ Mississippi pool#1 included DNA extracted from the following females: F3 (MS_1.F2, Copiah Co, MS, 17 Nov 2006, JGoddard); F5 (MS_3.F1, Copiah Co, MS, 28 Dec 2006, JGoddard); G4 (MS_7.F3, Copiah Co, MS, 29 February 2008, JGoddard); G6 (MS_8.F1, Copiah Co, MS, 19 November 2008, JGoddard) [16]. ^d^ Mississippi pool#2 included DNA extracted from the following females: F6 (MS_4.F1, Copiah Co, MS, 10 January 2007, JGoddard), F8 (MS_4.F3, Copiah Co, MS, 10 January 2007, JGoddard), and G11 (MS_6.F1, Copiah Co, 4 April 2007, JGoddard) [16].

**Table 2:** Oligonucleotides used in this study.

Taxon	Primer Name	Target Gene	Size (bp)	Primer Sequence (5′ → 3′)	Reference
*Anaplasma phagocytophilum*	ApMSP2f	Msp2 (major surface protein 2)	77	ATGGAAGGTAGTGTTGGTTATGGTATT	[23]
	ApMSP2r			TTGGTCTTGAAGCGCTCGTA	
*Ap Taqman probe*	ApMSP2p			/**HEX**/TGGTGCCAG/**ZEN**/GGTTGAGCTTGAGATTG/**IABkFQ**	
*Borrelia burgdorferi*	PC-1s	OspC	632	AATGAAAAAGAATACATTAAGTGCA	[24]
	PC-2a	OspC		TTAAGGTTTTTTTGGACTTTCTGC	
*Borrelia burgdorferi*	BBSCH31	FlaB (outer)	437	CACACCAGCATCACTTTCAGGGTCT	[25]
	BBSCH42	FlaB (outer)		CAACCTCATCTGTCATTGTAGCATCTTTTATTT	
*Borrelia burgdorferi*	FL59	FlaB (inner)	277	GCATTTTCAATTTTAGCAAGTGATG	[25]
	FL7	FlaB (inner)		TTTCAGGGTCTCAGGCGTCTT	
Candidatus *Cardinium*	CLOf	16S rRNA	1100	GCGGTGTAAAATGAGCGTG	[26]
	CLOr2	16S rRNA		ACCTMTTCTTAACTCAAGCCT	
Chalcidoideae	1775-COIF	COI	1047	CGA- ATAAATAATATAAGATTTTG	[27]
	2773-COI-R	COI		GGATAATCTCTATATCGACGAGGTAT	
*Flavobacterium*	qFlav16SF	16S rRNA	142	CGATGGATACTAGCTGTTGGG	This study
	qFlav16SR	16S rRNA		CGAATTAAACCACATGCTCCAC	
*Ixodes scapularis*	IsActin2F	Actin	271	GACCTGACCGACTACCTGATGAAG	This study
	IsActin2R	Actin		ATGCCGCACGATTCCATACC	
*Ixodiphagus hookeri*	401-F	COI	268	TTTAGAATATTTATTGATTCAGGGACT	[28]
	44-R			CTCCTGCTAAAACTGGTAAAGATAAT	
Legionellales	RLrpoB6f	rpoB	729	AGATGGTACGCSGGTTGATATCGT	[29]
	RLrpoB2r	rpoB		TTCCATTTGGTGATCGCCATC	
Nematode	Nema12SF	12S	450	GTT CCA GAA TAA TCG GCT A	[30]
	Nema12SR			ATT GAC GGA TG(AG) TTT GTA CC	
*Pseudomonas*	PA-GS-F	16S rRNA	618	GACGGGTGAGTAATGCCTA	[31]
	PA-GS-R	16S rRNA		CACTGGTGTTCCTTCCTATA	
*Pseudomonas*	16S qPCR_F	16S rRNA	143	TTGTCCTTAGTTACCAGCACG	This study
	16S qPCR_R	16S rRNA		ACCCTTTGTACCGACCATTG	
*Rickettsia*	r190.7	RompA	632	ATGGCGAATATTTCTCCAAAA	[32]
	r190.701	RompA		GTTCCGTTAATGGCAGCATCT	
	Rrgap2F	RompA	1664	CTACCTTGTACCTTGCTGAGCGAAA	This study
	Rrgap2R			AGCTTTGCAGCTAACGGCGCT	
*Rickettsia*	qRi16SF	16S rRNA	124	ACCTTACCAACCCTTGACATG	This study
	qRi16SR			GGACTTAACCCAACATCTCACG	
*Rickettsiella*	qRkla6F	16S rRNA	135	AAAGGAATTGACGGGGGCC	This study
	qRkla6F			CCTGTCTCTTGGTTCCTTTCGG	
*Wolbachia*	wsp 81F	wsp	610	TGG TCC AAT AAG TGA TGA AGA AAC	[33]
	wsp 691R	wsp		AAA AAT TAA ACG CTA CTC CA	

**Table 3:** Samples used for validation. All samples were collected from wild populations (from Central Pennsylvania). Samples were assayed as individuals (I) or pooled DNA (P) by PCR or qPCR. The housekeeping gene used for qPCR was *Ixodes scapularis* actin2.

			qPCR						PCR						
Population	Sex	I/P (N)	Ap MSP2	qRi	qRk5	qRk6	qPs	qFl	*FlaB*	*OspC*	*RompA*	*RLrpo*	*CLO*	*Nema*	*Chal*
Stormstown	Female	P (20)	☑	☑	☑	☑	☑	☑	☑	☑	☑	☑	☑	☑	☑
Stormstown	Males	P (20)	☑	☑	☑	☑	☑	☑	☑	☑	☑	☑	☑	☑	☑
BlueCourse	Males	P (15)	☑	☑	☑	☑	☑	☑	☑	☑	☑	☑	☑	☑	☑
BlueCourse	Female	P (18)	☑	☑	☑	☑	☑	☑	☑	☑	☑	☑	☑	☑	☑
APDS	Males	P (14)	☑	☑	☑	☑	☑	☑	☑	☑	☑	☑	☑	☑	☑
APDS	Female	P (16)	☑	☑	☑	☑	☑	☑	☑	☑	☑	☑	☑	☑	☑
Big Hollow	Males	P/I (15)	☑	☑	☑	☑	☑	☑	☑	☑	☑	☑	☑	☑	☑
		I (20)		☑		☑									
Big Hollow	Female	P (20)	☑	☑	☑	☑	☑	☑	☑	☑	☑	☑	☑	☑	☑
Galbraith	Female	P (20)	☑	☑	☑	☑	☑	☑	☑	☑	☑	☑	☑	☑	☑
Galbraith	Males	P (20)	☑	☑	☑	☑	☑	☑	☑	☑	☑	☑	☑	☑	☑
		I (20)		☑		☑									
Benner Springs	Males	P (20)	☑	☑	☑	☑	☑	☑	☑	☑	☑	☑	☑	☑	☑
Benner Springs	Female	P (20)	☑	☑	☑	☑	☑	☑	☑	☑	☑	☑	☑	☑	☑
SGL176	Female	P (20)	☑	☑	☑	☑	☑	☑	☑	☑	☑	☑	☑	☑	☑
SGL176	Males	P (20)	☑	☑	☑	☑	☑	☑	☑	☑	☑	☑	☑	☑	☑
Shaver’s Creek	Female	P/I (20)	☑	☑	☑	☑	☑	☑	☑	☑	☑	☑	☑	☑	☑
		I (20)		☑		☑									
Shaver’s Creek	Males	P (20)	☑	☑	☑	☑	☑	☑	☑	☑	☑	☑	☑	☑	☑
Julian, PA 2015	Females	P (16)	☑	☑	☐	☐	☐	☐	☐	☐	☐	☐	☐	☐	☐
Julian, PA 2015	Males	P (20)	☑	☑	☐	☐	☐	☐	☐	☐	☐	☐	☐	☐	☐
SGL333_2017	Females	P (20)	☑	☑	☐	☐	☐	☐	☐	☐	☐	☐	☐	☐	☐
SGL333_2017	Males	P (20)	☑	☑	☐	☐	☐	☐	☐	☐	☐	☐	☐	☐	☐
SGL333_2018	Females	P (50)	☑	☑	☐	☐	☐	☐	☐	☐	☐	☐	☐	☐	☐
SGL333_2018	Males	P (40)	☑	☑	☐	☐	☐	☐	☐	☐	☐	☐	☐	☐	☐
Howard, PA	Females	P (9)	☑	☑	☐	☐	☐	☐	☐	☐	☐	☐	☐	☐	☐
Howard, PA	Males	P (15)	☑	☑	☐	☐	☐	☐	☐	☐	☐	☐	☐	☐	☐
Lederer Park, State College	Females	P (21)	☑	☑	☐	☐	☐	☐	☐	☐	☐	☐	☐	☐	☐
Lederer Park, State College	Males	P (21)	☑	☑	☐	☐	☐	☐	☐	☐	☐	☐	☐	☐	☐

Abbreviations: ApMSP2 = *Anaplasma phagocytophilum* MSP2, Taqman; qRi = qPCR *Rickettsia* 16S rRNA, qRk5 or 6 = qPCR *Rickettsiella* 16S rRNA, qPs = Pseudomonas 16S rRNA, FlaB = *Borrelia burgdorferi* FlaB, Ospc *= Borrelia burgdorferi* ospC, RompA = *Rickettsia* outer membrane protein A, RLrpo = *Legionellales* rpoB, CLO = *Cardinium* 16S rRNA, *Nema = nematode primers, Chal = Chalcid* and *Ixodes hookeri* 18S rRNA.

**Table 4:** Genbank accession numbers used in phylogenetic analysis.

Taxon	Gene	Genbank Accession Number
*Rickettsia*	RompA	JFKF01000169.1, LN794217.1, CP003340.1, CP047359.1, CP003375.1, CP040325.1, CP001612.1, CP003308.1, CP001227.1, CP012420.1, CP000683.1, CP003342.1
*Rickettsiella*	rpoB	KP985349.1, KP985348.1, KP985347.1, KP985346.1, KP985354.1, KP985353.1, EF694042.1, JF288929.1, KP985351.1, KP985350.1, AP018005.1, KP985357.1, KP985356.1, KP985355.1
*Cardinium*	*16S rRNA*	AB241135.1, AB241129.1, GU731426.1, AB241131.1, AB241130.1, AY753169.1, MH057615.1, AY635291.1, AB001518.1, LN829689.2, JN204482.1, LC159289.1, AB241132.1, AF350221.1, AB116514.1, KX022134.1, AB506775.1, AB506773.1, AB506774.1, GQ206320.1, CP022339.1

## Supplementary Materials

The following are available online at https://www.mdpi.com/2075-4450/11/10/718/s1, Table S1: Read counts of amplicon sequence variants (ASV) by population. Figure S1: Stacked chart of the most abundant taxa sequenced from individual ticks using the bacterial 16S rRNA hypervariable region v6. Figure S2: Bacterial titers determined by qPCR of *Rickettsia*, *Pseudomonas*, and *Flavobacterium* from male and female individuals. Figure S3: Alpha diversity plots of v4 sequenced pools. Figure S4: Molecular Phylogenetic analysis of *Rickettsia* outer membrane protein (rompA) by Maximum Likelihood method. Figure S5: Molecular Phylogenetic Analysis of *Rickettsiella* rpoB gene by Maximum Likelihood. Figure S6: Molecular Phylogenetic Analysis of *Cardinium* 16S rRNA gene (1100 bp) by Maximum Likelihood. Figure S7: Bacterial titers of *Rickettsia* and *Rickettsiella* 16S rRNA gene from individual male and female ticks in select pools. External data: NCBI Sequence Bioproject Accession #:PRJNA669392.
