# Supplementary material for: Bacterial Communities of Ixodes scapularis from Central Pennsylvania, USA
Source: Insects. 2020 Oct 20;11(10):718. doi: 10.3390/insects11100718 (PMC7593946; doi:10.3390/insects11100718)
Supplement: Supplementary file 1 [file insects-11-00718-s001.zip › SupplFigs_highres/Supplemental_legends.pdf]

Supplemental Figure Legends

Figure S1. Stacked chart of the most abundant taxa sequenced from individual ticks using the bacterial 16S rRNA hypervariable region v6. Bacterial communities of males from Big Hollow and females from Shaver's Creek were more similar than either to females from Big Hollow. Big Hollow females were largely populated by Rickettsiaceae, while Big Hollow Males and Shaver's Creek Females were dominated by Pseudomonadaceae. The taxonomic assignment was done using the Greengenes reference database.

Figure S2. Bacterial titers determined by qPCR of *Rickettsia*, *Pseudomonas*, and *Flavobacterium* from male and female individuals. Data were normalized to a housekeeping gene *Ixodes scapularis* actin. Statistical significance determined by Mann-Whitney U. Females had significantly higher titers of *Rickettsia*, but significantly lower titers of *Pseudomonas* and *Flavobacterium* than males.

Figure S3. Alpha diversity plots of v4 sequenced pools. Colors represent populations, while shapes represent sex of the pools. Diversity indices of all populations show that the female pool of APD appears to be an outlier. We confirmed this using Grubb's analysis and it was omitted from subsequent analyses.

Figure S4. Molecular Phylogenetic analysis of *Rickettsia* outer membrane protein (rompA) by Maximum Likelihood method. Blue circles denote clones from Pennsylvania, the yellow circle denotes a clonal from Wisconsin (positive control), and the orange diamond denotes *Rickettsia buchneri*. The evolutionary history was inferred by using the Maximum Likelihood method based on the General Time Reversible model [38]. The bootstrap consensus tree inferred from 1000 replicates [39] is taken to represent the evolutionary history of the taxa analyzed [39]. Branches corresponding to partitions reproduced in less than 50% bootstrap replicates are collapsed. The percentage of replicate trees in which the associated taxa clustered together in the bootstrap test (1000 replicates) are shown next to the branches [39]. Initial tree(s) for the heuristic search were obtained automatically by applying Neighbor-Join and BioNJ algorithms to a matrix of pairwise distances estimated using the Maximum Composite Likelihood (MCL) approach, and then selecting the topology with superior log likelihood value. The analysis involved 16 nucleotide sequences. Codon positions included were 1st+2nd+3rd+Noncoding. All positions containing gaps and missing data were eliminated. There were a total of 1595 positions in the final dataset. Evolutionary analyses were conducted in MEGA7 [37].

Figure S5. Molecular Phylogenetic Analysis of *Rickettsiella* rpoB gene by Maximum Likelihood. Blue circles denote samples sequenced for this study and compared to sequences found on Genbank. The evolutionary history was inferred by using the Maximum Likelihood method based on the General Time Reversible model [38]. The bootstrap consensus tree inferred from 1000 replicates [39] is taken to represent the evolutionary history of the taxa analyzed [39]. Branches corresponding to partitions reproduced in less than 50% bootstrap replicates are collapsed. The

percentage of replicate trees in which the associated taxa clustered together in the bootstrap test (1000 replicates) are shown next to the branches [39]. Initial tree(s) for the heuristic search were obtained automatically by applying Neighbor-Join and BioNJ algorithms to a matrix of pairwise distances estimated using the Maximum Composite Likelihood (MCL) approach, and then selecting the topology with superior log likelihood value. The analysis involved 10 nucleotide sequences. Codon positions included were 1st+2nd+3rd+Noncoding. All positions containing gaps and missing data were eliminated. There were a total of 438 positions in the final dataset. Evolutionary analyses were conducted in MEGA7 [37].

Figure S6. Molecular Phylogenetic Analysis of *Cardinium* 16S rRNA gene (1100 bp) by Maximum Likelihood. The blue circle denotes the single isolate from this study and the orange diamond represents the closest sequence found on Genbank. The evolutionary history was inferred by using the Maximum Likelihood method based on the General Time Reversible model [38]. The bootstrap consensus tree inferred from 1000 replicates [39] is taken to represent the evolutionary history of the taxa analyzed [39]. Branches corresponding to partitions reproduced in less than 50% bootstrap replicates are collapsed. The percentage of replicate trees in which the associated taxa clustered together in the bootstrap test (1000 replicates) are shown next to the branches [39]. Initial tree(s) for the heuristic search were obtained automatically by applying Neighbor-Join and BioNJ algorithms to a matrix of pairwise distances estimated using the Maximum Composite Likelihood (MCL) approach, and then selecting the topology with superior log likelihood value. The analysis involved 22 nucleotide sequences. All positions containing gaps and missing data were eliminated. There were a total of 1070 positions in the final dataset. Evolutionary analyses were conducted in MEGA7[37].

Figure S7. Bacterial titers of *Rickettsia* and *Rickettsiella* 16S rRNA gene from individual male and female ticks in select pools. Data were normalized to a housekeeping gene *Ixodes scapulari*s actin. Statistical significance determined by Mann-Whitney-U and significance labels were produced in R. Females had significantly higher *Rickettsia* titers, but significantly lower *Rickettsiella* titers than males.
