# Supplementary material for: Bacterial Communities of Ixodes scapularis from Central Pennsylvania, USA
Source: Insects. 2020 Oct 20;11(10):718. doi: 10.3390/insects11100718 (PMC7593946; doi:10.3390/insects11100718)
Supplement: Supplementary file 1 [file insects-11-00718-s001.zip › SupplFigs_highres/FigureS2.pdf]

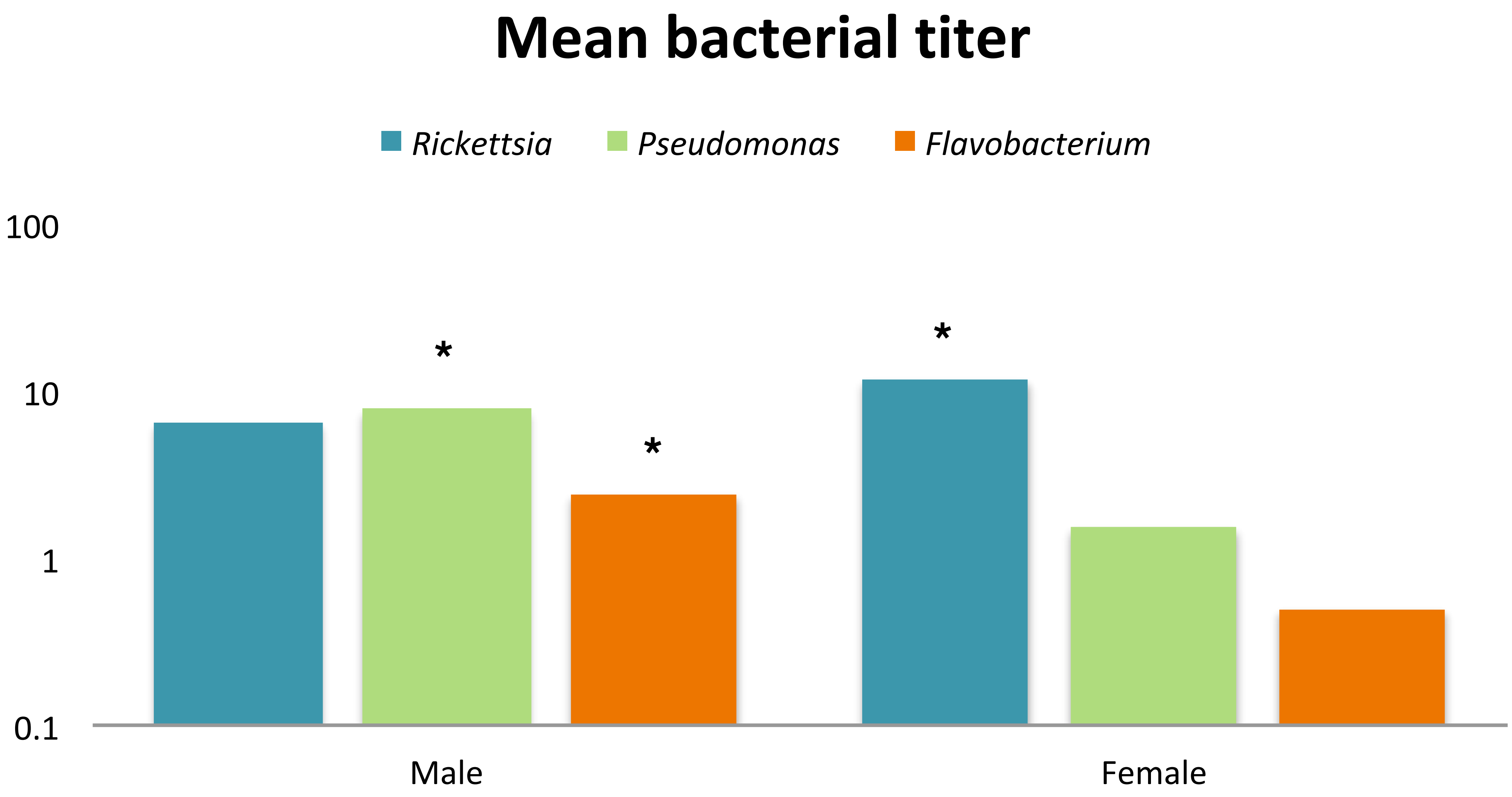

Rickettsia: Mann Whitney U= 87.5, z-score = 4.66816, p-value < .00001.

Pseudomonas: Mann Whitney U= 116.5, z-score = -4.15146, p-value < .00001.

Flavobacterium: Mann Whitney U= 113.5, z-score = -4.20491, p-value < .00001.
