# Supplementary figures and images for: Bacterial Communities of Ixodes scapularis from Central Pennsylvania, USA
Source: Insects. 2020 Oct 20;11(10):718. doi: 10.3390/insects11100718 (PMC7593946; doi:10.3390/insects11100718)

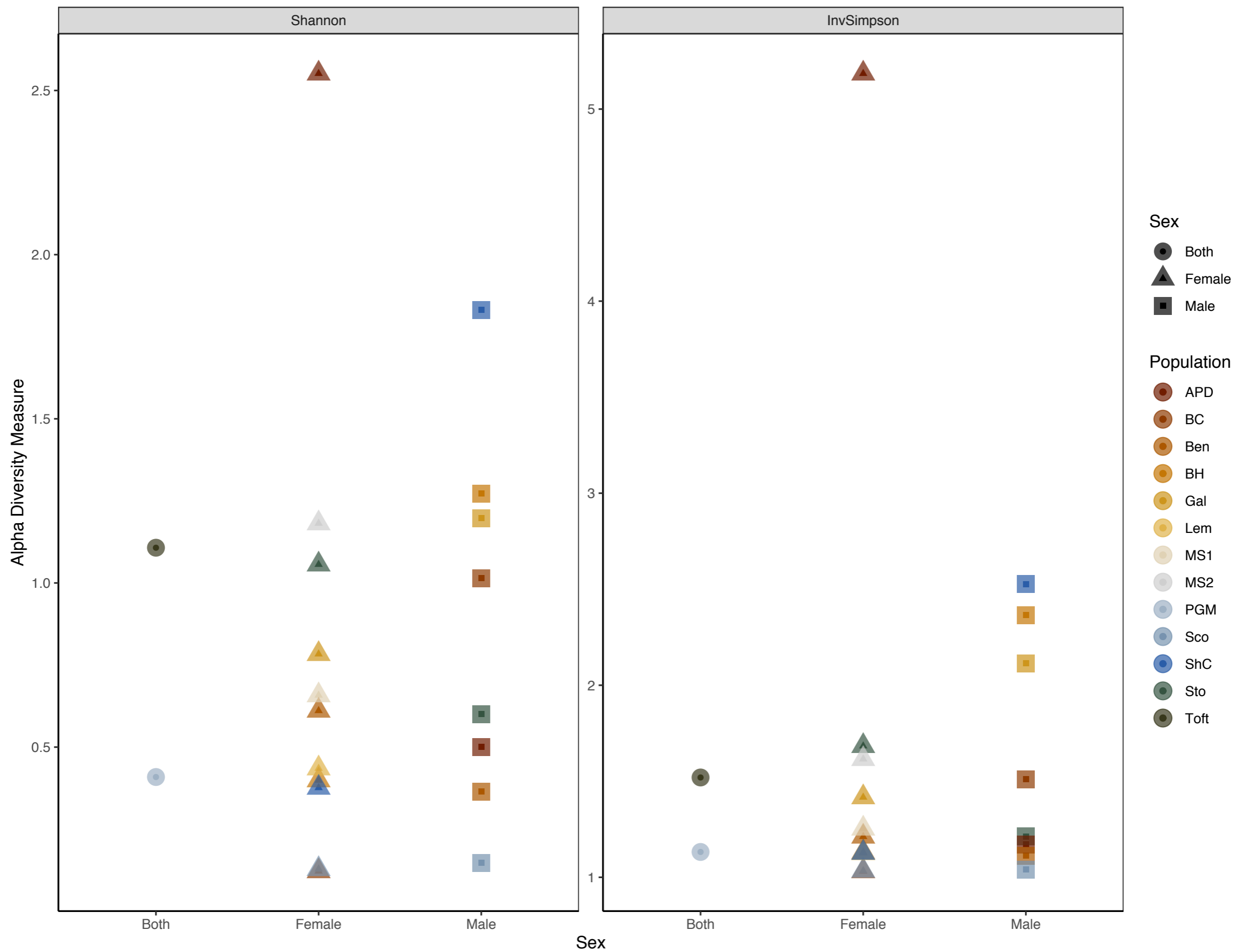

Supplement: Supplementary file 1 [file insects-11-00718-s001.zip › SupplFigs_highres/FigureS3.pdf]

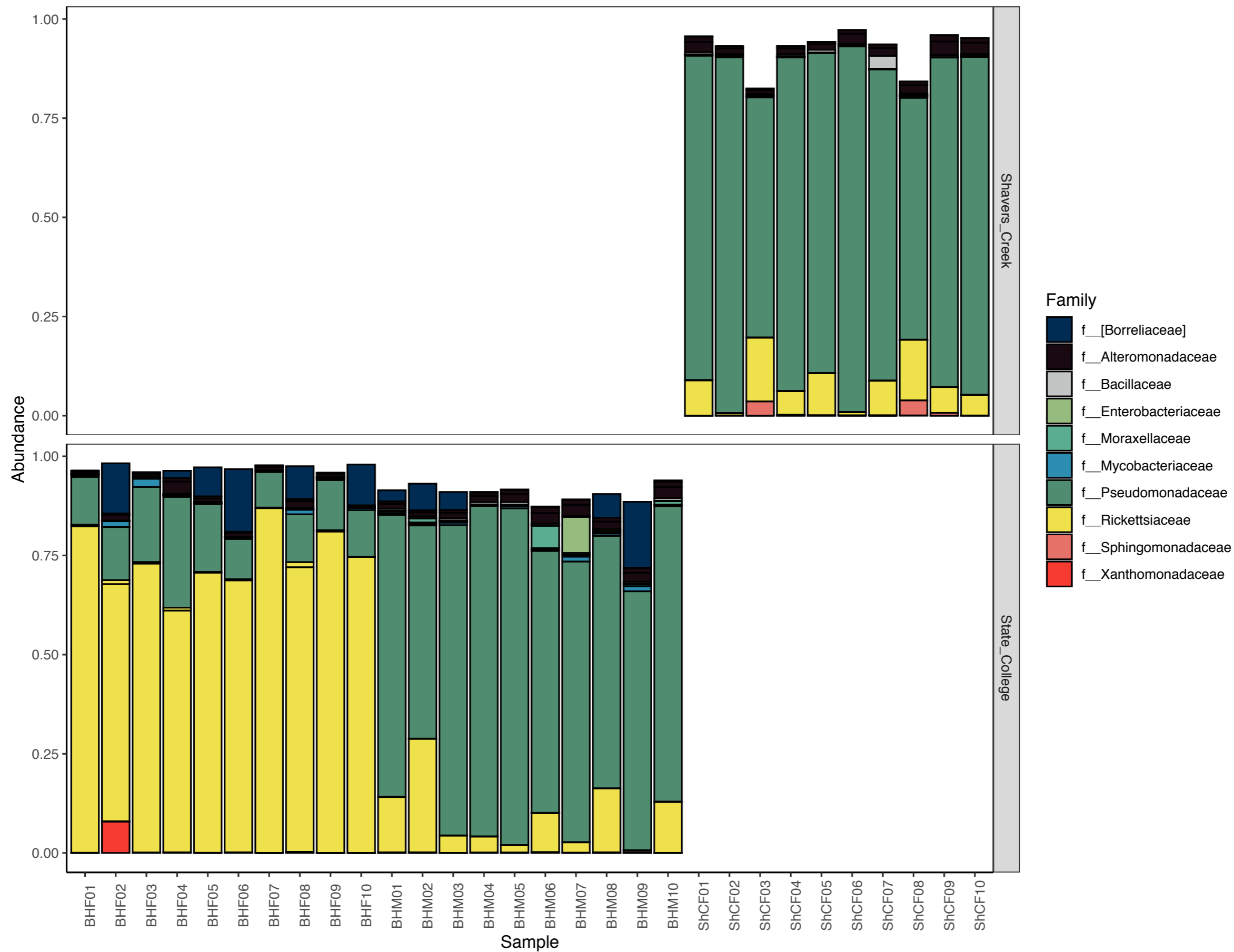

Supplement: Supplementary file 1 [file insects-11-00718-s001.zip › SupplFigs_highres/FigureS1.pdf]

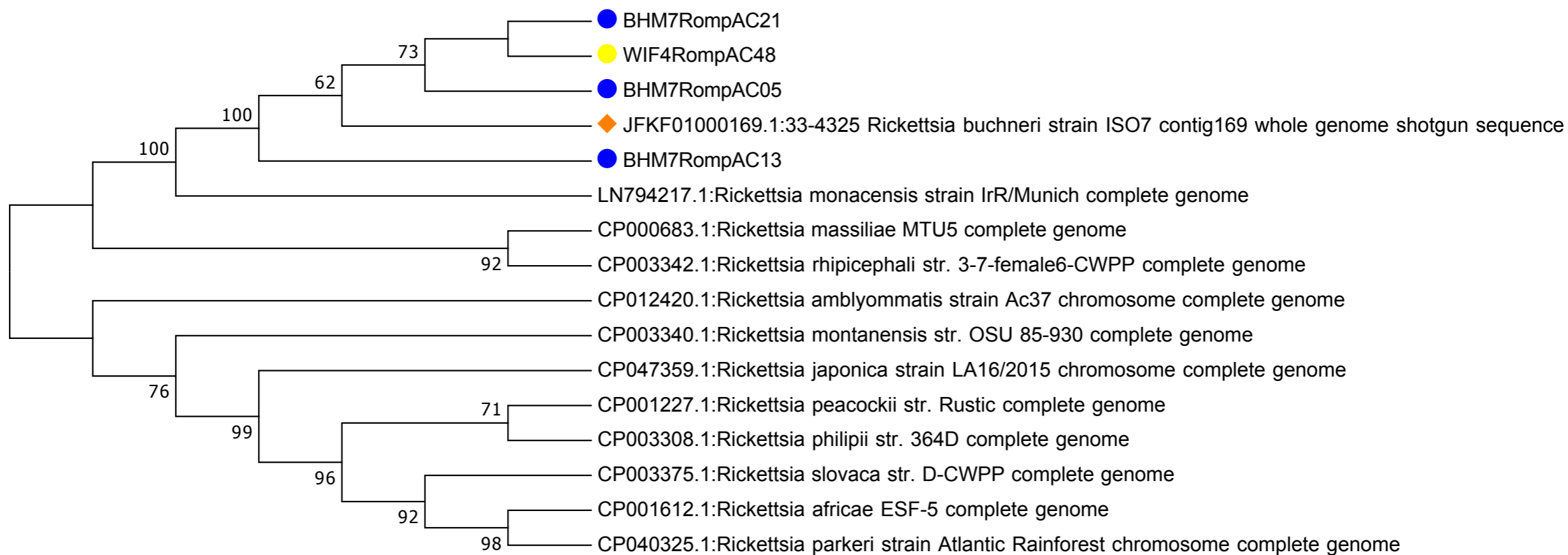

Supplement: Supplementary file 1 [file insects-11-00718-s001.zip › SupplFigs_highres/FigureS4.pdf]

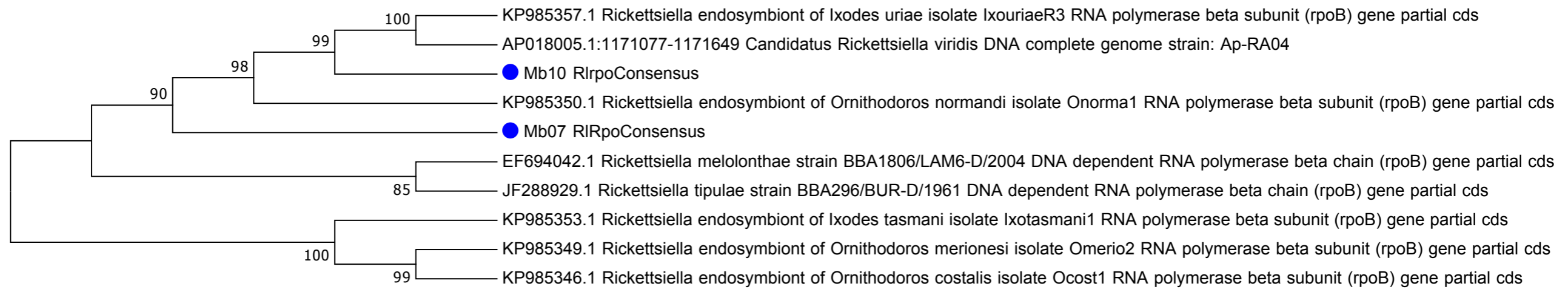

Supplement: Supplementary file 1 [file insects-11-00718-s001.zip › SupplFigs_highres/FigureS5.pdf]

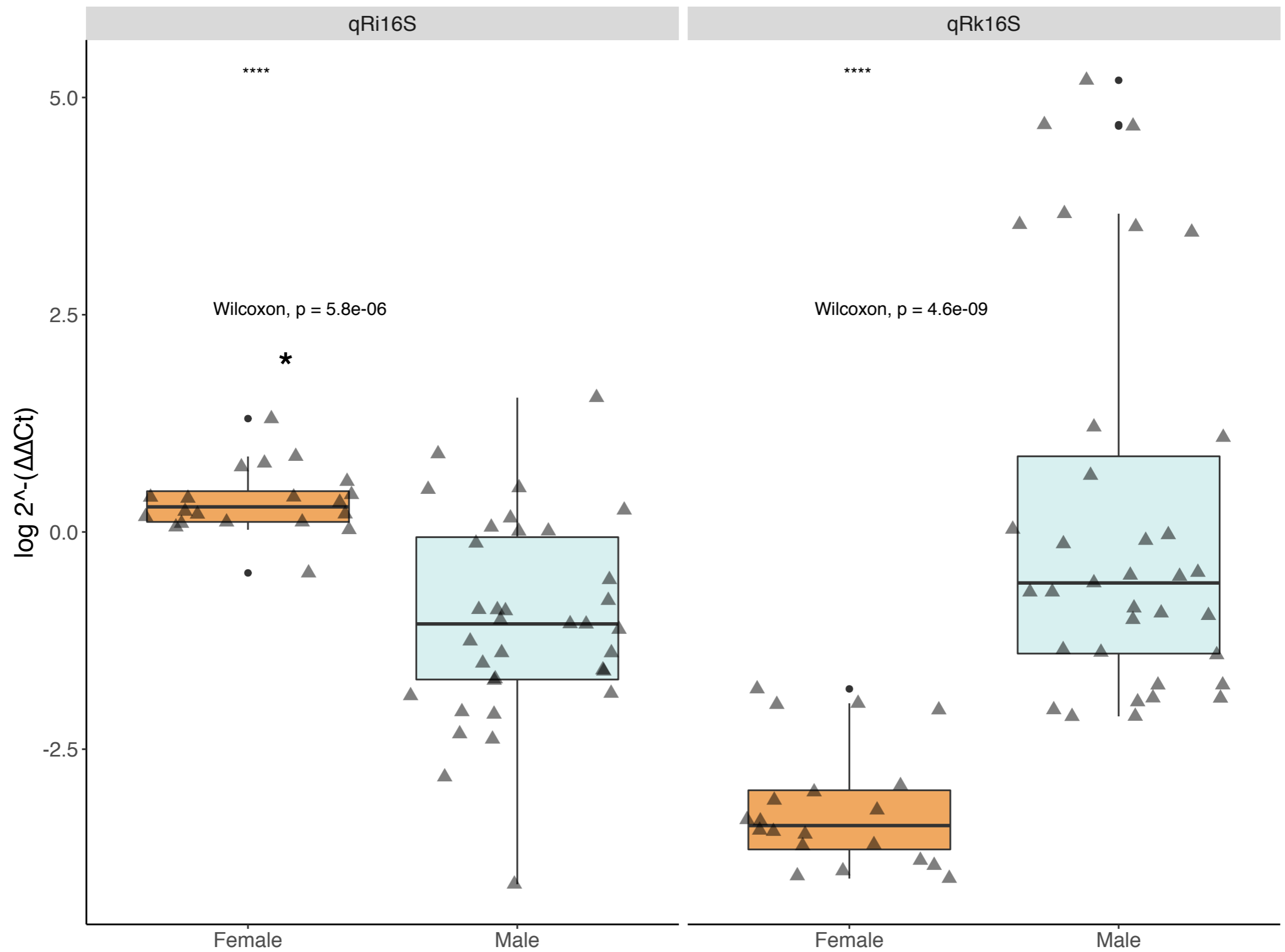

Supplement: Supplementary file 1 [file insects-11-00718-s001.zip › SupplFigs_highres/FigureS7.pdf]

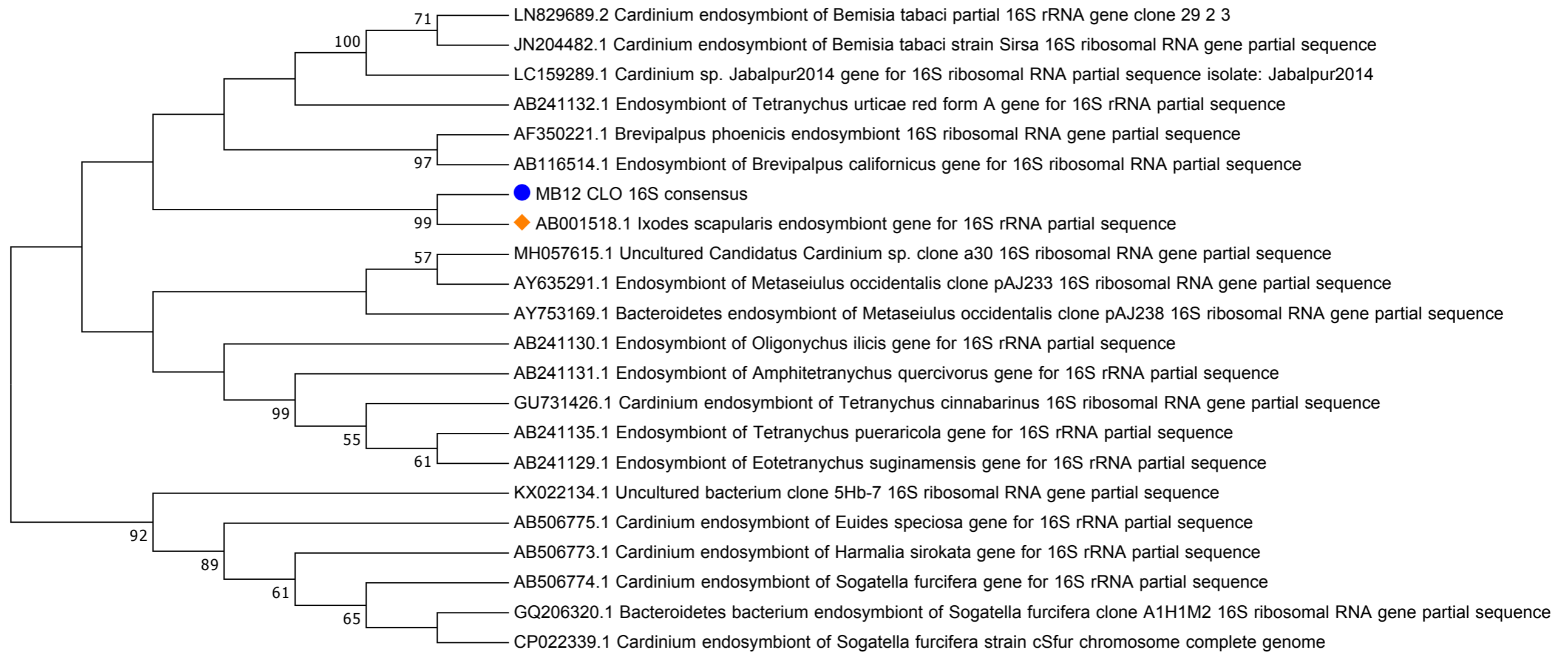

Supplement: Supplementary file 1 [file insects-11-00718-s001.zip › SupplFigs_highres/FigureS6.pdf]
